# Supplementary material for: Investigation of a Large Kindred Reveals Cardiac Calsequestrin (CASQ2) as a Cause of Brugada Syndrome
Source: Genes (Basel). 2024 Jun 21;15(7):822. doi: 10.3390/genes15070822 (PMC11275647; doi:10.3390/genes15070822)
Supplement: Supplementary file 1 [file genes-15-00822-s001.zip › genes-3033278-supplementary.pdf]

Table S1. Gene list associated to sudden arrhythmic death.

ABCA12	*AGTR1*	*CALM1*	*DNAJC19*	*GNAI2*	*HRC*	*LAT2*	*MYL2*	*RBM20*	*STX1A*	*VPS37D*
ABCB1	*AGTR2*	*CALM2*	*DNAJC30*	*GNB3*	*IKZF1*	*LDB3*	*MYL3*	*REN*	*SYNE1*	*VWF*
ABCC6	*AGXT2*	*CALM3*	*DPP6*	*GNPTAB*	*IL18*	*LDLR*	*MYOM1*	*RFC2*	*SYNE2*	*WAS*
ABCG5	*AKAP9*	***CASQ2***	*DSC2*	*GP1BA*	*INA*	*LDLRAP1*	*NAV1*	*RFFL*	*TBL2*	*WIPF1*
ABCG8	*AKT1*	*CAV3*	*DSG2*	*GP6*	*ITGA2*	*LEMD2*	*NCF1*	*RNF207*	*TBX3*	*ZNF365*
ABHD11	*ALG10B*	*CLCF1*	*DSP*	*GPC5*	*ITGB3*	*LIG3*	*NDRG4*	*RYR2*	*TBX5*	*ZNF385B*
ABO	*ANK2*	*CLCN1*	*DTNA*	*GPD1L*	*JUP*	*LIMK1*	*NEBL*	*SCD*	*TECRL*	*ZNF592*
ACAD9	*ANXA5*	*CLDN3*	*EIF4H*	*GPR37L1*	*KCNA4*	*LIN9*	*NOS1AP*	*SCN10A*	*TGFB3*	
ACADL	*AP1G2*	*CLDN4*	*ELN*	*GRIA1*	*KCND2*	*LIPC*	*NOS2*	*SCN1B*	*TH*	
ACADVL	*APOB*	*CLIP2*	*EMD*	*GSTA1*	*KCND3*	*LITAF*	*NOS3*	*SCN2B*	*THBS2*	
ACE	*APOE*	*COX8A*	*ENPP1*	*GSTM1*	*KCNE1*	*LMNA*	*NRG1*	*SCN3B*	*TKT*	
ACE2	*ATP2A2*	*CPB2*	*ERAL1*	*GSTP1*	*KCNE2*	*LPCAT1*	*P4HA2*	*SCN4A*	*TLR4*	
ACSL1	*B4GALNT3*	*CPT1A*	*ESR1*	*GSTT1*	*KCNE3*	*LRP6*	*PCSK9*	*SCN4B*	*TMEM270*	
ACSL3	*BAZ1B*	*CRLF1*	*EYA4*	*GTF2I*	*KCNH2*	*MAF*	*PGM1*	*SCN5A*	*TMEM43*	
ACTC1	*BAZ2B*	*CSMD2*	*F2*	*GTF2IRD1*	*KCNJ11*	*MBL2*	*PKP2*	*SERPINE1*	*TNNI3*	
ACYP2	*BCL7B*	*CSRP3*	*F5*	*GTF2IRD2*	*KCNJ2*	*METTL27*	*PLN*	*SLC25A45*	*TNNT2*	
ADRA2B	*BDKRB2*	***CTNNA3***	*FBN1*	*HAND1*	*KCNJ5*	*MLXIPL*	*PON1*	*SLC27A6*	*TPM1*	
ADRA2C	*BID*	*CXADR*	*FHL1*	*HCN4*	*KCNJ8*	*MLYCD*	*PRKAG2*	*SLC8A1*	*TOR1A*	
ADRB1	*BUD23*	*DDAH1*	*FHOD3*	*HEY2*	*KCNQ1*	*MMP3*	*PTEN*	*SLMAP*	*TRDN*	
ADRB2	*CACNA1C*	*DEGS2*	*FKBP1B*	*HLA-B*	*KCNQ1OT1*	*MYBPC3*	*PTPN22*	*SLN*	*TRPM4*	
AGPAT3	*CACNA2D1*	*DES*	*FKBP6*	*HLA-DQB1*	*KCTD1*	*MYH6*	*RAB3GAP1*	*SNTA1*	*TTN*	
AGT	*CACNB2*	*DMPK*	*GINS3*	*HLA-DRB1*	*KNG1*	*MYH7*	*RANGRF*	*SREBF2*	*USF1*	

Table S2 Variants prioritized in the analysis of the Brugada proband (VUS, Likely Benign and Benign).

GENE	Ref seq	CONSEQUENCE		ALLELE FREQUENCY GENOMES	ALLELE FREQUENCY EXOMES	ACMG Classification
CASQ2	NM_001232.3	Missense	c.532T>C, p.(Tyr178His)	not found	C*f* =0,0000014	VUS
CTNNA3	NM_013266.3	Missense	c.1561T>C, p.(Cys521Arg)	not found	C*f* = 0.00000636	VUS
HLA-DRB1	NM_002124.3	Missense	c.752G>A, p.(Arg251Lys)	A*f* =0.003063		VUS
HLA-DRB1	NM_002124.3	Missense	c.730G>A, p.(Ala244Thr)	A*f* =0.0010		VUS
NOS2	NM_000625.4	Missense	c.940C>T, p.(Arg314Cys)	T*f* =0.0002744	T*f*=0.000644	VUS
SYNE1	NM_182961.3	Missense	c.5710T>A, p.(Leu1904Met)	T*f* =0.00002	T*f* =0.00003	VUS
TOR1A	NM_000113.3	Missense	c.962C>T, p.Thr321Met	A*f*=0.001061.		VUS
TTN	NM_001267550.2	intron_variant, missense	c.76952T>C, p.(Val25651Ala)	C*f* =0.0009845	C*f* =0.001307	VUS/Likely Benign
TTN	NM_001267550.2	intron_variant, missense	c.107576T>C, p.(Met35859Thr)	G*f* =0.000634	G*f* =0.001939	VUS/Likely Benign
MLXIPL	NM_032951.2	Synonymous	c.1023C>T, (p.(Pro341=)	T*f* =0.00001974	T*f* =0.00007530	Likely Benign
NOS3	NM_000603.4	Synonymous	c.354G>C (p.(Pro118=)	A*f* =0.000045	C*f* =0.000090	Likely Benign
FBN1	NM_000138.4	Synonymous	c.306C>T, (p.(Cys102=)	T*f* =0.003722	T*f* =0.003739	Likely Benign
RAB3GAP1	NM_001172435.1	Synonymous	c.2463C>T, (p.(Phe821=)	T*f* = 0.0092		Benign
TLR4	NM_138554.4	Synonymous	c.1062A>G, (p.(Lys354=)	G*f* =0.003181		Benign
SCN4A	NM_000334.4	Synonymous	c.489C>G, (p.(Thr163=)	G*f* =0.008484	G*f* =0.009946	Benign
FHOD3	NM_001281740.2	Synonymous	c.3105A>C, p.Pro1035=)	C*f* =0.003447	C*f* =0.005074	Likely benign
TTN	NM_001267550.2	intron_variant, missense	c.107576T>C, p.(Met35859Thr)	C*f* =0.0018	C*f* =0.0022	Likely Benign
FHOD3	NM_001281740.2	Missense	c.1910G>A, p.(Arg637Gln)	A*f* =0.002105	A*f* =0.001745	Likely Benign
AKAP9	NM_005751.4	Missense	c.3827G>A, p.(Arg1276Gln)	A*f* =0.006347	A=*f* =0.009301	Likely Benign
NOS2	NM_000625.4	Missense	c.3052C>A, p.(Arg1018Ser)	T*f* =0.000128		Likely Benign
SCN5A	NM_000335.5	splice_region_variant|intron_variant	c.2788-6C>T	T*f* =0.042414	T*f* =0.032575	Likely benign
